# Comparative Molecular Analysis of Cancer Behavior Cultured In Vitro, In Vivo, and Ex Vivo

**DOI:** 10.3390/cancers12030690

**Published:** 2020-03-14

**Authors:** Nicholas R. Hum, Aimy Sebastian, Sean F. Gilmore, Wei He, Kelly A. Martin, Aubree Hinckley, Karen R. Dubbin, Monica L. Moya, Elizabeth K. Wheeler, Matthew A. Coleman, Gabriela G. Loots

**Affiliations:** 1 Physical and Life Sciences Directorate, Lawrence Livermore National Laboratory, Livermore, CA 94550, USA; hum3@llnl.gov (N.R.H.); sebastian4@llnl.gov (A.S.); gilmore24@llnl.gov (S.F.G.); he4@llnl.gov (W.H.); martin249@llnl.gov (K.A.M.); hinckley3@llnl.gov (A.H.); coleman16@llnl.gov (M.A.C.); 2 School of Natural Sciences, University of California Merced, Merced, CA 94550, USA; 3 Engineering Directorate, Lawrence Livermore National Laboratory, Livermore, CA 94550, USA; dubbin1@llnl.gov (K.R.D.); moya3@llnl.gov (M.L.M.); wheeler16@llnl.gov (E.K.W.); 4 Department of Biochemistry and Molecular Medicine, University of California Davis, Sacramento, CA 95817, USA

**Keywords:** syngeneic culture, PDX, spheroid, tumoroid, monolayer culture, RNA-seq, TNBC, 4T1, single-cell RNA-seq, EMT

## Abstract

Current pre-clinical models of cancer fail to recapitulate the cancer cell behavior in primary tumors primarily because of the lack of a deeper understanding of the effects that the microenvironment has on cancer cell phenotype. Transcriptomic profiling of 4T1 murine mammary carcinoma cells from 2D and 3D cultures, subcutaneous or orthotopic allografts (from immunocompetent or immunodeficient mice), as well as ex vivo tumoroids, revealed differences in molecular signatures including altered expression of genes involved in cell cycle progression, cell signaling and extracellular matrix remodeling. The 3D culture platforms had more in vivo-like transcriptional profiles than 2D cultures. In vivo tumors had more cells undergoing epithelial-to-mesenchymal transition (EMT) while in vitro cultures had cells residing primarily in an epithelial or mesenchymal state. Ex vivo tumoroids incorporated aspects of in vivo and in vitro culturing, retaining higher abundance of cells undergoing EMT while shifting cancer cell fate towards a more mesenchymal state. Cellular heterogeneity surveyed by scRNA-seq revealed that ex vivo tumoroids, while rapidly expanding cancer and fibroblast populations, lose a significant proportion of immune components. This study emphasizes the need to improve in vitro culture systems and preserve syngeneic-like tumor composition by maintaining similar EMT heterogeneity as well as inclusion of stromal subpopulations.

## 1. Introduction

Cancer has a major impact on society and poses a significant financial burden globally [1]. The American Cancer Society predicts that ~1.7 million new cases of cancer will be diagnosed this year and ~20% of all deaths will be cancer associated. While the overall cancer death rate has dropped steadily since 1991, this decline is mostly due to changes in lifestyle (smoking cessation) and early detection (breast and colorectal). Cancer remains a challenging health burden as the second leading cause of death, in both men and women, with death rates continuing to rise for liver, pancreatic, endometrial, and brain cancers [2]. The past two decades have expanded our understanding of the mutational landscape and signal transduction pathways that drive tumorigenesis, guiding the development of more effective therapies and improving survival outcomes for numerous cancer types [3]; however, advances have remained slow for patients at later stages of the disease or those with highly aggressive cancer subtypes [4]. Most significantly, >95% of new therapies that exhibit superior performance in animal models fail in the clinic due to therapeutic inefficacy or unwarranted toxicity [5].

In order to improve future pre-clinical cancer models, a thorough understanding of molecular changes underlying cancer cell behavior in vivo or ex vivo is essential [3]. However, the vast accumulation of gene expression data generated from clinical tumor samples is primarily collected via bulk tumor RNA sequencing or microarray analysis. These datasets represent the transcriptional output of all stromal and malignant cells combined, making it difficult to deconvolute deviations accounted solely by cancer cell response [6]. As a result of this technical challenge, heterogeneity found in a pre-clinical screening has classically been underrepresented and initial screens are typically performed on in vitro cultures of solely cancer cells.

Conventional in vitro two-dimensional (2D) culture of human cancer cell lines has been fundamental in the study of cancer biology as well as for screening and evaluating therapeutic efficacies of anticancer agents. This approach promotes fast growth, ample area for adherence to substrates, and uniform access to nutrients and growth factors. Overall, 2D cancer models generate highly reproducible results, but lack the cellular complexity and extracellular matrix (ECM) associated with solid tumors [3]. Three-dimensional (3D) cancer cell culture methods that generate spheroids, organoids, or cells embedded in various ECM compositions have been shown to display enhanced cell–cell interactions [7,8] yet are only superficially accessible to nutrients and diffusible drugs. In general, 3D cultures exhibit enhanced cellular heterogeneity and preserve characteristics apparent in the original tumor [7]. However, these culture methods currently fail to incorporate the complex heterogenous cell composition and diffusion of nutrients or drugs that occur in vivo.

Animal models such as patient-derived xenografts (PDXs) allow for aspects of in vivo tumor progression and incorporate components of the physiologically relevant microenvironment. In recent years, PDXs have been increasingly used to expand our understanding of factors affecting tumor growth, drug response and metastasis [8]; however, the absence of a complete immune system allows human tumors to evolve in a murine-specific manner and these models are not useful for testing many immune-based therapies [9]. Allograft rodent models utilizing mouse-derived cancer cell lines or genetic mouse models capable of spontaneous generation of tumors represent the most clinically relevant cancer models due to the presence of in vivo conditions as well as a full repertoire of stromal cell types, including immune cells. Yet less than 8% of animal model findings are successfully translated to clinical cancer trials [5,10]. Additionally, in vivo culturing is hindered by labor and financial burdens posed by the lengthy process associated with establishing tumor engraftment and generating cohorts for experimentation.

Heterogenous ex vivo organoid cultures from primary clinical tumors (tumoroids) have gained considerable traction in recent years due to ease of culturing and the ability of tumoroids to maintain stromal cellular complexity. Tumoroids permit a faster culturing method, amenable to multiplexing a wider range of analysis tools in a pre-clinical setting. Several studies have shown promising results of tumoroid cultures mimicking histological morphology and drug responses across multiple cancer types [11,12,13,14,15]. However, a thorough analysis of the shifting stromal cell populations and the resulting effects on cancer cell behavior as a product of the culturing method have yet to be thoroughly investigated.

In order to understand the relationship and limitations of various culturing approaches, we examined the transcriptome of a triple-negative mammary carcinoma murine cell line, 4T1, in various culturing environments (in vitro, in vivo, or ex vivo). The 4T1 cells are well suited, as they closely mimic human breast cancer [16] and can form syngeneic tumors in fully immune-competent mice. Transcriptional analysis identified distinct molecular profiles corresponding to in vitro and in vivo culturing conditions while ex vivo tumoroids exhibited molecular characteristics associated with both approaches. Several key biological processes [cell cycle progression, ECM remodeling, cell signaling, and epithelial-mesenchymal transition (EMT) progression] critical to tumor progression also varied across culturing conditions. Tumoroids were found to represent the most similar in vitro method to tumors established in syngeneic mice. However, despite the high similarity in cancer cell behavior, tumoroid composition, as assessed by single-cell RNA sequencing (scRNA-seq), displayed significant shifts in stromal subpopulations after 5 days of ex vivo culture; changes that may play important roles in modulating cancer cell behavior. Faithful recapitulation of the transcriptional behavior, EMT heterogeneity, and stromal heterogeneity will be critical to understanding key deficiencies in existing culturing systems as well as educating future engineered tumor platforms in fully recreating endogenous tumor architecture and response to therapy.

## 2. Results

### 2.1. Cancer Cell Transcriptome is Dictated by Culture Conditions

**Figure 1:**
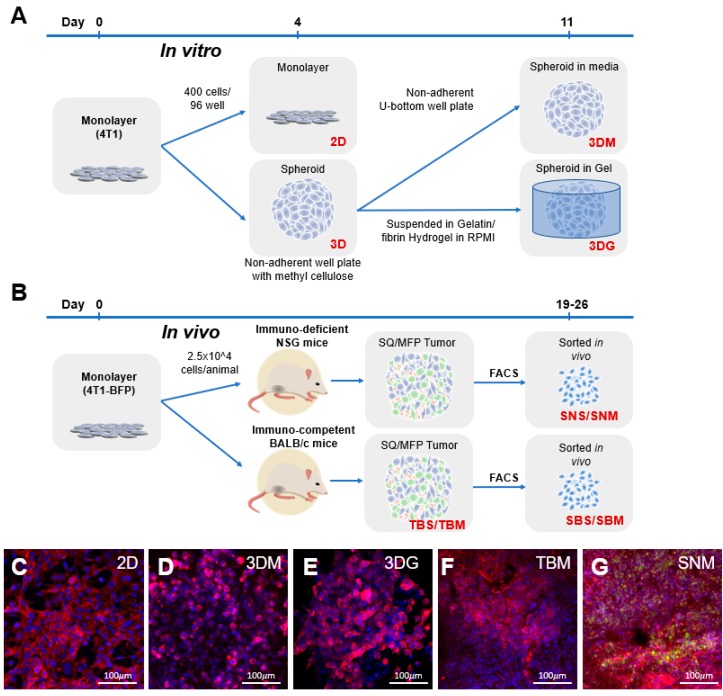
Experimental overview. (**A**) The 4T1 in vitro samples originated from low passage number, subconfluent, monolayer cultured cells seeded at 400 cells/well in 96 wells into tissue culture-treated flat-bottom plates or non-adherent U-bottom plates. Following 4 days of culture, monolayer (**C**) RNA was collected, and spheroids continued to be cultured in wells containing media (**D**) or cast into a gelatin/fibrin hydrogel (**E**) for an additional 7 days prior to RNA isolation. (**B**) In vivo tumor samples were generated by injection of subconfluent, monolayer 4T1-BFP cultures into mammary fat pad (MFP) or subcutaneously into (SQ) back flank locations in immunodeficient (NSG) or BALB/c mice. Tumors were isolated following a 19–26-day growth period yielding tumors ranging from 70 to 140 mm^3^. RNA samples were processed from bulk (**F**) or BFP^+^ cancer cell populations isolated by fluorescently activated cell sorting (FACS) (**G**). Blue: nuclear DAPI staining; red: phalloidin staining actin filaments; green: BFP expression.

RNA sequencing was performed on 4T1 cells grown in different culture modalities to determine molecular phenotypes driven by the culture environment (Figure 1). In vitro methodologies profiled included a conventional 2D 4T1 monolayer culture on polystyrene tissue culture-treated flasks (Figure 1C), spheroids cultured in non-adherent well plates in culture media for 7 days following a 4-day initial spheroid formation (3DM) (Figure 1D) or spheroids encapsulated in a gelatin-fibrin hydrogel (3DG) (Figure 1E) for 7 days post-spheroid formation. In vivo methodologies required generation of 4T1 cells engineered to express blue fluorescent protein (BFP) to enable the purification of-4T1-BFP^+^ cells from the heterogenous tumor environment using fluorescently activated cell sorting (FACS) (Supplementary Figure S1). Transcriptional profiles of cancer cells isolated from primary tumors of immunodeficient (NSG) mice and syngeneic, immune-competent (BALB/c) mouse models were analyzed in this study. While malignant cells inoculated into the tissue of origin is ideal for reproducing native stromal environments, subcutaneous administration of tumor cells offers a more technically reproducible and simple approach to introducing cancer cells in vivo. Here, we comprehensively examine the transcriptional effects imparted by localization of primary tumors in both orthotopic (mammary fat pad (MFP)) and subcutaneous (back flank (SQ)) sites (Figure 1B).

Conventionally, cancer cells are cultured in a monolayer (2D). For this reason, transcriptional profiles of alternate 4T1 culturing conditions were referenced to 2D culture to identify differentially expressed genes (DEGs) in each platform. The sorted Balb/c-derived MFP (SBM) culture represents the most clinically relevant model of tumor derived cancer cells, because the cancer cells are purified from an orthotopic site, and tumors are grown in an immune competent strain of mice. Henceforth, successful recapitulation of native cancer cell behavior will be in comparison to SBM samples. Histologically, in vivo 4T1-BFP^+^ tumors had more densely packed cells than 4T1 cells cultured in spheroids (Figure 1C–G) and labeled 4T1 cells were surrounded by other stromal cell types (Figure 1G, 4T1 (green); actin (red); DAPI (blue)).

**Figure 2:**
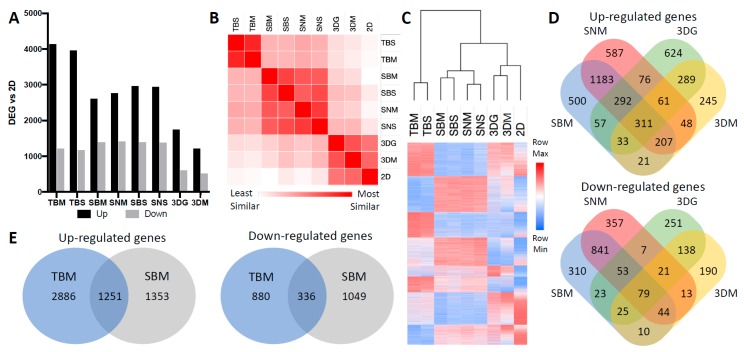
Transcriptomic variability of cancer cells under different culturing conditions. (**A**) Quantity of differentially expressed genes (DEGs) compared to 2D samples. DEGs were defined as greater than 2-fold change and FDR < 0.05 (n ≥ 4). Cultured condition abbreviations: TBM: whole *T*umor from *B*ALB/c *M*ammary fat pad; TBS: whole *T*umor from *B*ALB/c *S*ubcutaneous back flank; SBM: *S*orted *B*ALB/c *M*ammary fat pad; SBS: *S*orted *B*ALB/c *S*ubcutaneous; SNM: *S*orted *N*SG *M*ammary fat pad; SNS: *S*orted *N*SG *S*ubcutaneous; 3DG: *3D* spheroids in hydro*G*el; 3DM: 3D spheroids in *M*edia; 2D: *2D* monolayer. (**B**) Similarity matrix based on whole transcriptome similarity of average expression values within each condition. Similarity differences calculated using Euclidean distance. (**C**) Heat map and dendrogram of whole transcriptome based on normalized logarithmic average expression values within each condition and hierarchal clustering of samples based on Euclidean distance. (**D**) Venn diagrams representing overlapping up- and down-regulated DEGs from 4T1 cultured from immunocompetent tumors, immunodeficient tumors, spheroids in gel, and spheroids in media compared to monolayer culture. (**E**) Venn diagrams representing overlapping up- and down-regulated DEGs from BALB/c MFP whole tumor vs. BALB/c MFP-sorted 4T1 compared to monolayer culture.

The 4T1 MFP (whole tumor BALB/c mammary fat pad (TBM)) (Figure 1F) and SQ (whole tumor BALB/c subcutaneous (TBS)) transcriptional profiles were most divergent from 2D cultured cells but showed high similarity to each other (Figure 2A–C; Supplementary Table S1). Whole tumor bulk RNA comparisons to 2D cells produced the highest number of DEGs, likely due to stromal cellularity (Figure 2A). However, these genes did not correlate with changes exclusive to 4T1 cells, since 4T1-BFP^+^ sorted from these tumors only shared 1251/2604 (48%) up-regulated and 336/1385 (24%) down-regulated transcripts (Figure 2E).

In vivo sorted BFP^+^ 4T1 cells from BALB/c MFP (SBM) and SQ (SBS), NSG MFP (SNM) and SQ (SNS) tumors clustered most closely with themselves. However, 3D spheroids induced a greater level of in vivo-like transcriptional level (Figure 2B,C; Supplementary Tables S2 and S3). Interestingly, 4T1-BFP^+^ cells derived from orthotopic MFP and SQ tumors were highly similar to each other in both immunocompetent and immunodeficient mice with minimal variability in the quantity and identity of genes differentially expressed compared to 2D (Supplementary Table S2). MFP and SQ tumors shared 79% and 83% of DEGs in immune -competent and -deficient mice, respectively. As expected, immune-deficiency did drive unique gene expression changes within 4T1 cells, where only 1993/2604 up-regulated (75%) and 1017/1385 down-regulated (73%) DEGs were shared by 4T1-BFP^+^ cells grown in BALB/c (SBM) and those grown in NSG mice (SNM) (Figure 2D). Interestingly, 4T1-BFP^+^ cells in syngeneic mice up-regulate a diverse set of genes associated with cellular processes indicative of differentiation and interactions with the surrounding microenvironment (Table 1). Furthermore, ECM organization, immune response, cell signaling, in addition to polarization and migration of cells were functional categories enriched in all in vivo conditions (Table 1). Relative to in vivo-derived cancer cells, cells cultured in monolayer promoted a set of cellular processes involved in multiple aspects of cellular proliferation (Table 1, Supplementary Figure S2) such as DNA synthesis, RNA processing, protein translation, as well as cell cycle progression, suggesting that 2D cultured cells encourage proliferation.

### 2.2. Culturing Condition Affects Cancer Cell Behavior Critical to Cancer Progression

Cancer requires a successive acquisition and management of critical cell behaviors in order to promote disease progression. Here, we further examined differential expression of biological processes associated with tumor progression to understand the imparted behavioral impact from varying culturing conditions. Dysregulation of cell cycle progression is a hallmark of cancer initiation and a target for numerous chemotherapeutic treatments [17]. However, alteration in cell metabolism is also required for cancer progression and includes extracellular matrix remodeling [18], cell–cell communication via secreted cell signaling [19,20], as well as transitioning from an epithelial to a mesenchymal cell state also known as EMT [21].

**Figure 3:**
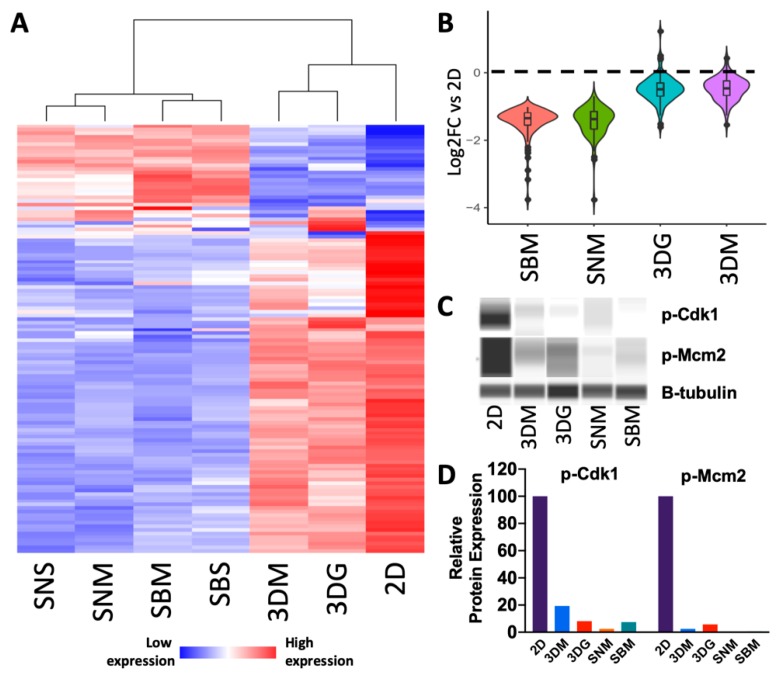
Cell cycle progression genes are up-regulated in cells cultured in monolayer culture. (**A**) Heat map of differentially expressed cell cycle genes (Supplementary Table S4) relative to 2D culture depicting increased down-regulation as culturing complexity increases (n = 4–5). (**B**) Violin plot depicting magnitude of down-regulation and distribution of SBM vs. 2D up-regulated cell cycle progression genes across other culturing conditions relative to 2D culture. (**C**) Pseudogel representation of protein levels of phosphorylated cell cycle genes (Cdk1 and Mcm2) from 4T1 cells cultured across multiple conditions. (**D**) Quantification of protein levels relative to β-tubulin.

Genes associated with proliferation and cell division were highly expressed in 2D, but these transcripts were least abundant in 4T1 cells purified from in vivo tumors (Figure 3A). This involved 88 genes down-regulated in SBM relative to 2D that are associated with cell cycle and includes several cyclin transcripts (*Ccna2*, *Ccnb1*, *Ccnd2*, *Ccne2*) (Figure 3A, Supplementary Table S4). Both 3DG and 3DM showed modest expression of cell cycle genes, whereas cells sorted from in vivo tumors significantly repressed this gene set (Figure 3B). Genes robustly expressed in monolayer also showed enrichment in biological processes that promote growth and cell division including DNA synthesis, RNA processing, and ribosomal translation. For example, cyclin-dependent kinase substrates involved in mitotic functions (*Ccp110, Npm1, Cdc6, Cdc25a*) and DNA replication (*Fen1, Orc1, Orc2*) were significantly up-regulated in 2D relative to in vivo. However, 22 cell cycle-associated genes including cyclin-dependent kinase inhibitor 1 a (*Cdkn1a*), a regulator of cell cycle progression, and members of the ubiquitin-proteasome system (*Ubb*, *Ubc, Psmb8*, *Psmb9*, *Psmb10*, *Psme1*, *Psme2*) were expressed at higher levels in vivo (Figure 3A; Supplementary Table S4). Specifically, immunoproteases (*Psmb8-10*) associated with proliferative human embryonic stem cells (hESC) [22] were amongst the most highly expressed cell cycle genes in vivo, suggesting up-regulation of genes associated with stemness, in vivo. Cell cycle processes are not only regulated at the transcriptional level but involve tightly controlled translational and post-translational regulation. Up-regulated levels of phosphorylated Cdk1 (a regulator of progression into mitosis) and phosphorylated Mcm2 (a regulator of S-phase progression) were observed in monolayer culture. Decreased expression of these cell cycle genes was identified in 3D cultured cells and very low expression was found in in vivo cultured cells (Figure 3C,D).

**Figure 4:**
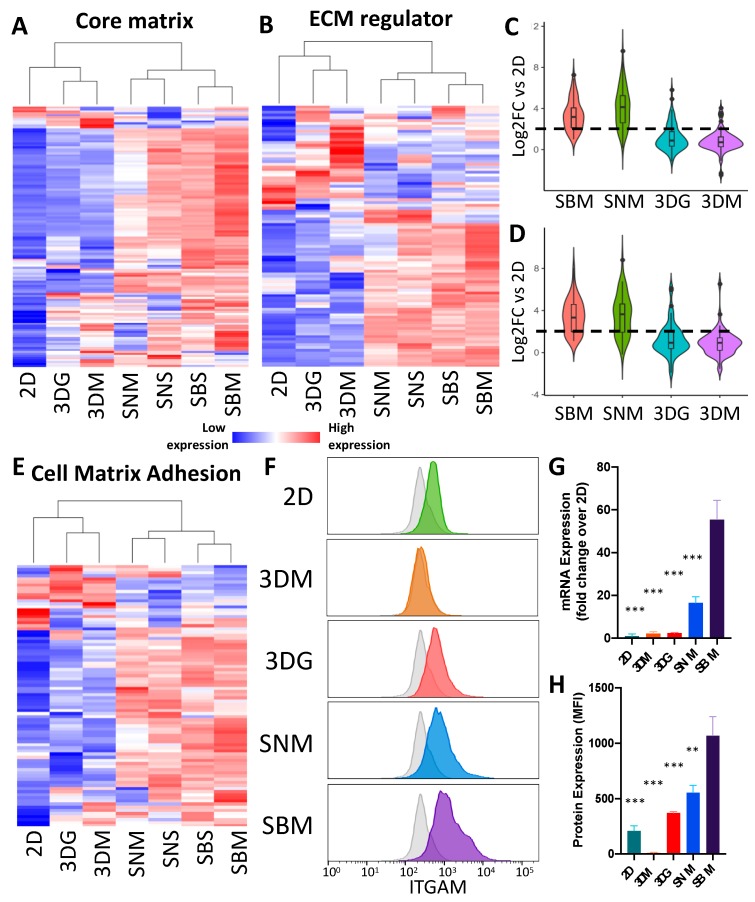
Extracellular matrix organization genes are up-regulated in cells cultured in 3D and in vivo conditions. Heat maps of differentially core matrix genes (**A**) (Supplementary Table S5) and ECM regulator genes (**B**) (Supplementary Table S6) relative to 2D culture. Expression values represented are an average of 4–5 replicates. Violin plots depicting magnitude of up-regulation and distribution of SBM vs. 2D up-regulated core matrix genes (**C**) and ECM regulator genes (**D**) across other culturing conditions relative to 2D culture. Violin plot depicting magnitude of up-regulation and distribution of SBM vs. 2D up-regulated core matrix genes across other culturing conditions relative to 2D culture. (**E**) Heat map of differentially expressed cell matrix adhesion genes (Supplementary Table S7) relative to 2D culture (n = 4–5). (**F**) Histogram of representative *Itgam* abundance of single cancer cells cultured in different methods. Grey shaded plots represent unstained controls. (**G**) mRNA expression levels of *Itgam* in 4T1 cells under differing culturing condition. Expression levels normalized to 2D culture ± SEM. (n = 4–5). (**H**) Bar graph of flow cytometric analysis of ITGAM protein expression showing up-regulation in 3DG and in vivo conditions. Protein expression values represent background (unstained control) subtracted median fluorescent intensity of cancer cells ± SEM; (n = 4). (** *p* value < 0.001; *** *p* value < 0.0001 relative to SBM).

Structuring and remodeling of the tumor ECM and surrounding tissue is an essential facet of tumor initiation, extravasation and intravasation that allow the disease to progress [18]. The 4T1 cells grown in monolayer had low expression of core matrix genes including collagens, *Eln, Bgn, Dcn, fibulins and fibrillins* (Figure 4A; Supplementary Table S5); genes associated with ECM regulation (Figure 4B; Supplementary Table S6) and cell matrix adhesion (Figure 4E; Supplementary Table S7). In contrast, these genes were robustly expressed in vivo and in 3D culture, which expressed significantly higher levels of ECM-associated genes. Although ECM-related genes were up-regulated in all in vivo and 3D models, a large number of ECM genes showed the highest level of up-regulation in syngeneic mice (SBM), with decreased levels in the immune deficient animals (SNM) (Figure 4C,D). Expression was further decreased under 3D culturing conditions (Figure 4C,D). Spheroids cultured in hydrogel did encourage moderate up-regulation of both core matrix and ECM regulating genes above levels in spheroids cultured in media and thus expression levels were more similar to the in vivo conditioned cell behavior.

ECM regulators highly expressed in vivo included protease gene families [matrix metalloproteinases (*Mmp1a, Mmp2, Mmp3, Mmp12 and Mmp13*), cathepsins (*Ctsf, Ctsk, Ctso* and *Ctss*), and ADAMTS (*Adamts2, Adamts6, Adamts12, Adamts14*)] as well as ECM crosslinking genes (lysyl oxidases)—many of which were also up-regulated in 3D cultures compared to 2D, but this up-regulation was more modest than in SBM samples (Supplementary Table S6). Several *Mmps* (Mmp15, Mmp24), however, were found to be down-regulated in 3D and in vivo tumors, relative to 2D culture. Two integrin genes (*Itgam* and *Itga4*) found to be differentially regulated were used to validate protein expression via flow cytometry. Itgam protein expression increased with culturing complexity, where 2D cultured cells expressed the lowest amounts, while SBM expressed the greatest amount (Figure 4F, H), correlating with transcriptional data (Figure 4G), with the exception of the 3DM sample, where the protein could not be detected (Figure 4F,H). This deviation in Itgam protein levels from transcriptional expression suggests subsequent translational regulation.

**Figure 5:**
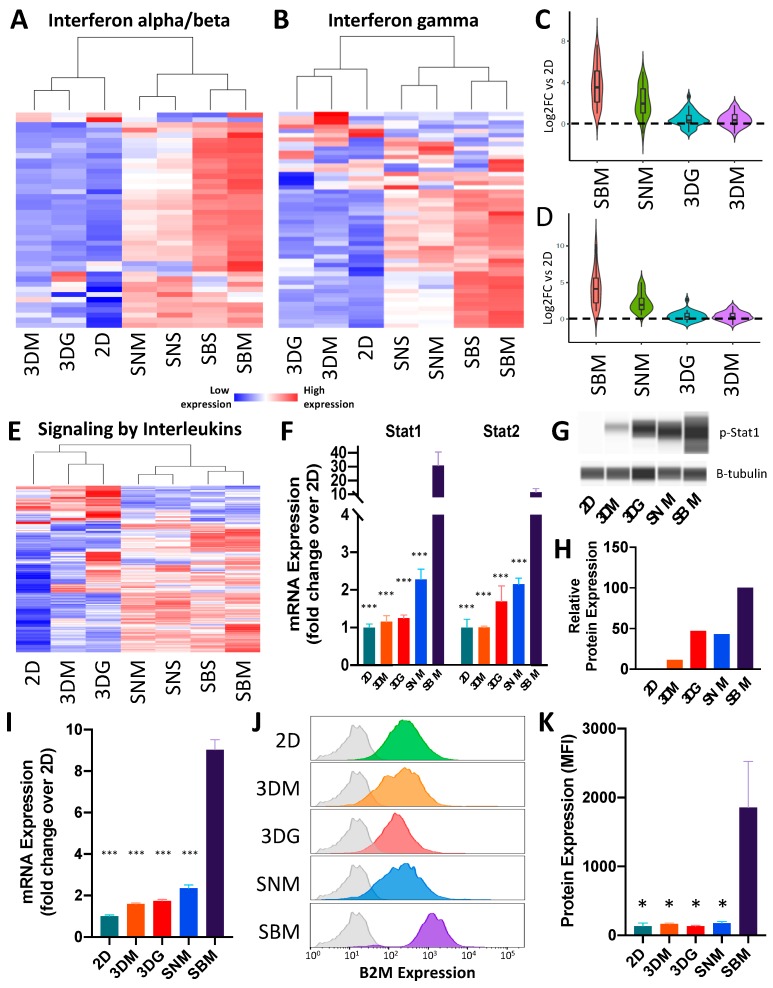
Cell signaling is highly up-regulated under syngeneic culturing conditions. (**A**) Heat map of differentially expressed interferon alpha/beta signaling genes (Supplementary Table S8) relative to 2D culture (n = 4–5). (**B**) Heat map of differentially expressed interferon gamma signaling genes (Supplementary Table S9) relative to 2D culture (n = 4–5). (**C**) Violin plot depicting magnitude of up-regulation and distribution of SBM vs. 2D up-regulated interferon alpha/beta signaling genes across other culturing conditions relative to 2D culture. (**D**) Violin plot depicting magnitude of up-regulation and distribution of SBM vs. 2D up-regulated interferon gamma signaling genes across other culturing conditions relative to 2D culture. (**E**) Heat map of differential signaling by interleukin genes (Supplementary Table S10) relative to 2D culture (n = 4–5). (**F**) mRNA expression levels of *Stat1* and *Stat2* in 4T1 cells under different culturing conditions. Expression levels normalized to 2D culture ± SEM (n = 4–5). (**G**) Pseudogel representation of protein levels of phosphorylated Stat1 from 4T1 cells cultured across multiple conditions. (**H**) Quantification of p-Stat1 protein levels relative to β-tubulin. (**I**) mRNA expression levels of B2M in 4T1 cells under differing culturing conditions. Expression levels normalized to 2D culture ± SEM; n = 4–5. (**J**) Histogram of representative Beta-2-microglobulin (B2M) abundance of single cancer cells cultured in different methods. Grey shaded plots represent unstained controls. (**K**) Bar graph of flow cytometric analysis of B2M protein expression showing up-regulation in BALB/c MFP. Protein expression values represent background (unstained control) subtracted median fluorescent intensity of cancer cells ± SEM (n = 4). (* *p* value < 0.05; *** *p* value < 0.0001 relative to SBM).

Many of the behavioral changes of cancer cells including proliferation, motility, and immune interaction could be mapped back to activation of cell signaling pathways [19]. Unsupervised clustering identified several cell-signaling pathways (RAS, TNF, PI3K-AKT, MAPK, interferon and interleukins) that were significantly up-regulated under in vivo conditions but showed a modest increase in 3D conditions (Supplementary Figures S2A and S3). Specifically, interferon alpha and beta (IFNα/β) (Figure 5A, Supplementary Table S8), interferon gamma (IFNγ, Supplementary Table S9) (Figure 5B), and signaling by interleukins (Figure 5E, Supplementary Table S10) were significantly up-regulated in cancer cells isolated from immune-competent mice (SBM). This effect was significantly reduced in immunodeficient mice (SNM) and only minimal up-regulation was measured in cancer cells cultured in 3D for both IFNα/β (Figure 5C) and IFNγ (Figure 5D) signaling. Surprisingly, both IFNα/β receptor subunits (IFNAR1 and IFNAR2) showed modest up-regulation (1.47 ± SD 0.165 and 1.84 ± SD 0.93 respectively) across all conditions; however, significant up-regulation of downstream targets was observed in cancer cells isolated only from syngeneic tumors including IRF transcription factors (*Irf1*, *Irf2*, *Irf4*, *Irf7*, *Irf8*, *Irf9*) as well as interferon target genes (*Ifi27, Ifi35, Ifit1, Ifit3, Ifitm1, Ifitm3, Isg15, Isg20*).

IFNγ pathway genes also showed greatest expression in cancer cells cultured under syngeneic conditions, with modest up-regulation in immunodeficient in vivo conditions and minimal up-regulation in 3D conditions, relative to 2D (Figure 5B). Although IFNγ receptors were not up-regulated in SBS and SBM conditions, Beta-2-microglobulin (B2M), a downstream target of IFNγ signaling, was 9.03 fold up-regulated in BALB/c tumors relative to 2D cultures, a > 3.5X increase above all other condition (Figure 5I), suggesting that cancer cells significantly up-regulate the IFNγ pathway under syngeneic conditions. Quantification of protein levels analyzed via flow cytometry confirmed that B2M expression was promoted by a fully competent immune system, with minimal activation in NSG mice or 3D conditions (Figure 5J,K). Consistent with interferon signaling, interleukin signaling-associated genes were only weakly stimulated in 3D cultures, both in gel or in media, and underlie the necessity of stromal and immune cell signaling for recapitulation of in vivo cancer cell behavior.

STAT complexes serve as critical transcription factors mediating gene expression in response to both IFNα/β and IFNγ signaling [23]. Transcriptionally, both *Stat1* and *Stat2* were most highly expressed in syngeneic conditions (Figure 5F). However, phosphorylation leads to translocation of STAT complexes into the nucleus where they bind DNA and activate target genes. To examine activation of both these signaling pathways, we quantified phosphorylated Stat1 (p-Stat1) levels as a metric of interferon signaling in 4T1 cells. Consistent with transcriptional levels, a significant increase in pStat1 levels was observed only in BALB/c-derived cancer cells (Figure 5H).

**Figure 6:**
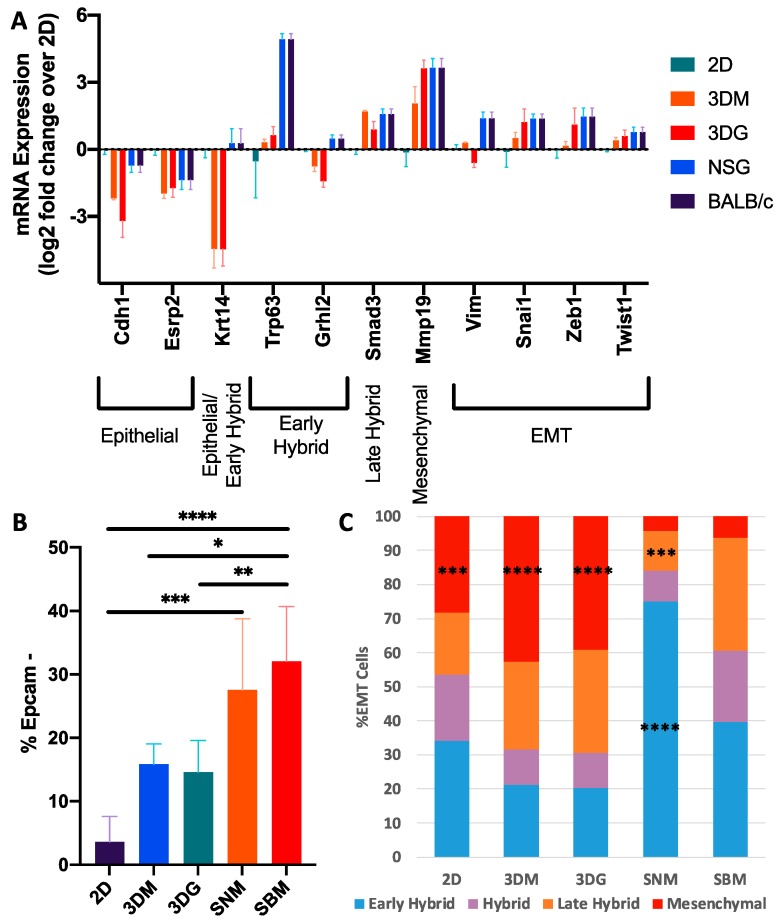
The 4T1 cells in vivo reside in multiple transitional epithelial-to-mesenchymal transition (EMT) states. (**A**) The 4T1 log2 expression of EMT-related genes under different culturing methods relative to 2D culture. Error bars ± SEM. (**B**) Proportion of cells lacking Epcam expression based on flow cytometric analysis (n = 4–7). Error bars ± SD. (**C**) Distribution of EMT cells across hybrid EMT states induced by the culturing method (n = 4–7). * *p* < 0.05, ** *p* < 0.0005, *** *p* < 0.0005, and **** *p* < 0.0001 relative to SBM.

Critical pathways that indicate the transition from epithelial-to-mesenchymal phenotype (EMT) include suppression of proliferation via cell cycle progression, increased ECM remodeling, and stimulation of cell signaling [21]. Recently, Pastushenko et al. identified the existence of multiple cancer cell subpopulations associated with different EMT states being classified in distinct stages: from epithelial to completely mesenchymal states, passing through intermediate hybrid states which were described as early hybrid, hybrid, late hybrid and mesenchymal states [24]. To determine the cellular EMT states induced by various culturing conditions, we first examined the expression levels of genes known to be associated with EMT (Figure 6A). As expected, 4T1 monolayers expressed high levels of epithelial markers *Cdh1* and *Esrp* and low levels of mesenchymal markers *Mmp19* and *Vim.* In addition, 4T1 monolayers expressed low levels of EMT associated transcription factors *Snai1*, *Zeb1* and *Twist1*. In contrast, EMT markers *Krt14*, *Trp63*, and *Grhl2*, which have been recently shown to correspond to an early hybrid state [25], were significantly up-regulated solely in in vivo conditions. However, late hybrid *Smad3* and mesenchymal marker *Mmp19* were significantly up-regulated in both 3D and in vivo conditions suggesting that tumors in vivo reside in more diverse EMT hybrid states. Suppression of proliferation via cell cycle progression, increased ECM remodeling, and stimulation of cell signaling pathways, are hallmarks of a transition from epithelial-to-mesenchymal phenotype (EMT) [21]. To determine the cellular EMT states induced by various culturing conditions, we first examined the expression levels of genes known to be associated with EMT (Figure 6A) [24]. As expected, 4T1 monolayers expressed high levels of epithelial markers *Cdh1* and *Esrp* and low levels of mesenchymal markers *Mmp19* and *Vim.* In addition, 4T1 monolayers expressed low levels of EMT associated transcription factors *Snai1*, *Zeb1* and *Twist1*. In contrast, EMT markers *Krt14*, *Trp63*, and *Grhl2*, which have been recently shown to correspond to an early hybrid state [24], were significantly up-regulated solely in in vivo experimental conditions. However, late hybrid *Smad3* and mesenchymal marker *Mmp19* were significantly up-regulated in both 3D and in vivo conditions, suggesting that tumors in vivo reside in more diverse EMT hybrid states under the culturing conditions described.

Flow cytometric analysis was utilized to further probe the heterogeneity of EMT states induced by 2D and 3D culturing and gated based on previously described methods [24]. Loss of epithelial phenotype was primarily examined through the loss of Epcam expression. Consistent with the transcriptional data, monolayers largely maintained epithelial status with only 3.6% of cells undergoing EMT. The 3D cultured samples increased the frequency of cells undergoing EMT. However, this increase was not statistically significant from monolayer culture. Additionally, there was no difference observed as a result of encapsulation into the hydrogel (15.8% in media and 14.6% in gel). In vivo conditions induced a significant increase in the abundance of these cells (27.5% SNM, 32.1% SBM), with no significant differences between immunodeficient and immunocompetent hosts (Figure 6B).

Cells undergoing EMT were further analyzed for the frequency of hybrid states based on the presence of cell surface markers CD51, CD61, and CD106. Transitional hybrid EMT states were classified into progressively more mesenchymal subpopulations as follows: (1) early hybrid EMT (triple negative and CD106+), (2) hybrid EMT (CD51+, CD51+/CD106+), (3) late hybrid EMT (CD51+/CD61+), and (4) Mesenchymal (CD51+/CD61+/CD106+). In vitro culturing promoted a greater abundance of mesenchymal cells than in vivo. Additionally, in vivo cultured cells possessed an increased abundance of early and hybrid populations relative to in vitro samples. Stromal composition in vivo was associated with EMT distribution as shown by the increased abundance of early hybrid and decreased abundance in late hybrid EMT cells in immunodeficient tumors relative to syngeneic tumors. (Figure 6C).

### 2.3. Ex Vivo Tumoroids Inclusive of Stromal Cells Preserve In Vivo Behavior

**Figure 7:**
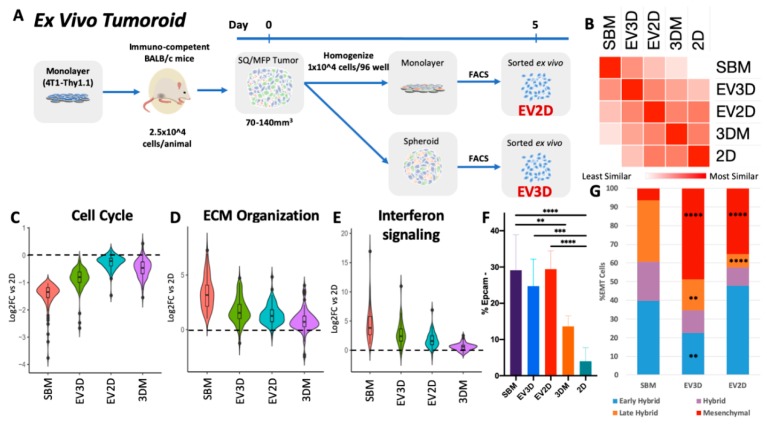
Ex vivo tumoroid culture encourages in vivo-like cancer cell behavior. (**A**) Ex vivo tumoroid culture experimental design. (**B**) Similarity matrix of 4T1 transcriptomic profiles across in vivo, ex vivo, and in vitro conditions. (**C**) Violin plot of magnitude of differential expression of cell cycle DEGs of SBM vs. 2D across culturing conditions. (**D**) Violin plot of magnitude of differential expression of ECM organization DEGs of SBM vs. 2D across culturing conditions. (**E**) Violin plot of magnitude of differential expression of interferon signaling DEGs of SBM vs. 2D across culturing conditions. (**F**) Flow cytometric analysis of 4T1 cells undergoing EMT following 5 days of ex vivo culturing. (**G**) Abundance of hybrid EMT states following ex vivo culturing. Tumoroid conditions n = 6 from three independent tumors. * *p* < 0.05, ** *p* < 0.0005, *** *p* < 0.0005, and **** *p* < 0.0001 relative to SBM.

As shown by altered cell signaling processes and EMT distribution at the RNA and protein level, stromal complexity affects cancer cell behavior; however, in vitro research with cell lines is classically performed exclusive of stromal cells. In order to examine the contribution of stromal cells in vitro, we created ex vivo tumoroid cultures from tumor cell homogenates cultured for 5 days as a monolayer or spheroids prior to FACS sorting of cancer cells for transcriptomic analysis (Figure 7A). Incorporation of stromal components increased 4T1 global transcriptomic similarity to syngeneic SBM conditions where ex vivo monolayer (EV2D) and spheroid (EV3D) were more similar to in vivo derived 4T1 cells compared to any other 4T1 in vitro method (Figure 7B). Interestingly, in silico hierarchal clustering of 4T1 transcriptomes discriminated in vitro from in vivo conditions (Figure 2C). However, incorporation of tumoroid cultures aligned EV3D closer to in vivo conditions, while the EV2D were more similar to the in vitro cultures (Figure 7B).

Examination of ex vivo gene regulation of critical cancer processes previously profiled further supported global transcriptome analysis that EV3D tumoroids best retain in vivo characteristics compared to EV2D or homogenous in vitro cultures in cell cycle, ECM, and cell signaling (Figure 7C–E). EV2D samples had improved in vivo gene expression similarity in ECM and cell signaling genes. However, cell cycle genes reverted to a monolayer-like expression level after 5 days of ex vivo culture. The presence of stromal cells in tumoroid cultures also maintained higher rates of cancer cells EMT diversity (Figure 7F) regardless of 2D or 3D culture. However, ex vivo culturing rapidly encouraged EMT cells into a more mesenchymal state and reduced the frequency of cells present in transitional EMT states, as both tumoroid conditions yielded more EMT cells in a mesenchymal state (EV3D: 49%, EV2D: 35%) in similar abundance to 4T1 cultured in 3D alone. Late hybrid populations were also significantly lower (EV3D: 17%, EV2D: 7%) than in syngeneic tumors. Surprisingly, EV2D maintained a higher rate of early hybrid EMT cells (48%) while EV3D frequency was reduced (22.5%) (Figure 7G).

**Figure 8:**
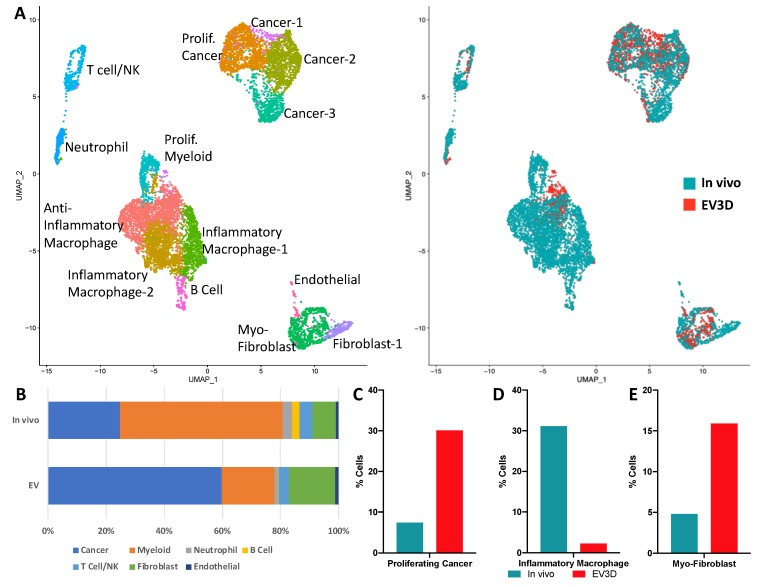
Single-cell RNA-seq analysis of 4T1 tumor and tumoroid cultures. (**A**) Merged uniform manifold approximation and projection (UMAP) of identified cells from a syngeneic tumor (in vivo) and 5-day ex vivo tumoroid (EV3D) cultures. (**B**) Cell type abundance from original tumor and tumoroids. (**C**) Abundance of proliferating cancer cells (Epcam^+^/Mki67^+^) in tumor/tumoroid. (**D**) Abundance of inflammatory macrophages (Ptprc^+^/CD14^+^/Il1b^+^) in tumor/tumoroid. (**E**) Abundance of myofibroblast (Thy1^+^/Dcn^+^/Acta2) in tumor/tumoroid.

The inclusion of stromal heterogeneity into tumoroid cultures recapitulated aspects of syngeneic cancer cell behavior yet tumoroid cultures still evolved features associated with in vitro culturing of 4T1 cells alone. We hypothesized that this rapid change may be in part due to loss of specific stromal subpopulations. In order to investigate differences in tumor and tumoroid composition, scRNA-seq was utilized to compare cellular heterogeneity in 3D tumoroid cultures (Figure 8A). Cancer cell populations more than doubled over the 5 days of ex vivo culture, increasing the ratio of tumor cells from 24.7% in the harvested tumor to 59.7% in the tumoroids (Figure 8B). The expansion of cancer cell populations ex vivo was validated via flow cytometry and observed in both 2D and 3D conditions (Figure S5). More specifically, ex vivo culturing encouraged the expansion of Epcam^+^/Mki67^+^ cancer cells from 7.4% to 30.1% of the total cells (Figure 8C). Fibroblasts comprised 8.0% of the original tumor and ex vivo culturing expanded this population to 16.0% of the tumoroid. In particular, the myofibroblast (Thy1^+^/Dcn^+^/Acta2^+^ (αSMA)) fraction which comprised 4.8% of all cells in the original tumor increased to 15.9% of the tumoroid cells (Figure 8E).

The 4T1 syngeneic tumors had high immune (Ptprc/CD45^+^) infiltration, with a high abundance of myeloid-derived neutrophils (3.4%) and monocytes/macrophages (55.9%) (Figure 8A). During ex vivo culture, these populations were reduced to 1.2% and 18.1%, respectively. Furthermore, the loss in monocyte/macrophage abundance was largely attributed to loss of inflammatory macrophages (Ptprc^+^/CD14^+^/Il1b^+^) that were reduced from 27.9% to 0.3% of the tumor, post-ex vivo culturing (Figure 8D), while anti-inflammatory and proliferating myeloid cells maintained comparable ratios in tumoroid cultures ~17.0% and ~3.4%, respectively. T-cell/Natural killer populations remained in similar proportions (~4%) following ex vivo culture as well as endothelial cells comprising ~1% of the cells in both conditions. Lastly, B-cell populations comprised 2.6% of cells in vivo yet decreased ex vivo to 0.3% in tumoroids (Figure 8A).

## 3. Discussion

Our study demonstrates the importance of culture platform selection in pre-clinical and research applications. Comparisons of purified cancer cells from different in vitro conditions (2D, 3D in media or hydrogel) and in vivo (immune- deficient and -competent mice, orthotopic and subcutaneous) studies revealed that cancer cell phenotype is highly influenced by its microenvironment. Conventional transcriptomic analysis of whole tumor bulk RNA analysis can lead to erroneous interpretations of tumor biology; we found that only 39% of transcripts significantly changed in cancer cells purified from orthotopic immune-competent tumors (SBM) overlapped with genes significantly changed when whole tumor bulk RNA was performed (Figure 2E). Furthermore, 2402 significantly changed SBM transcripts were distinct from TBM significant changes. These results suggest that bulk tumor RNA analysis overlooks a vast molecular space that can be potentially interrogated for contribution to cancer progression, invasion, metastasis and response to therapy.

Upon examination of solely cancer cell transcriptional behavior, cellular processes prioritized were closely linked to functions promoted by different culturing conditions. For example, cancer cells grown in monolayers favored rapid proliferation, and this behavior was corroborated molecularly by significant up-regulation of cell cycle progression genes in addition to metabolic processes that synthesize DNA, RNA, and proteins. The 2D cultured cells also maintained a high level of cellular homogeneity, where ~96% of 4T1 cells persisted in an epithelial cell state. Carcinomas of different tissue origin, including lung and gastric, have been documented to undergo EMT, as characterized by a loss in epithelial behaviors like cell polarity and cell–cell adhesion [25] and a decreased proliferation in cells up-regulating EMT genes [26].

Unlike cancer cells cultured in monolayers, cancer cells in 3D down-regulate proliferative processes, while up-regulating genes involved in ECM organization and cell adhesion. Spheroids in gel showed minimal changes in gene expression profiles compared to spheroids in media alone, and some of the differences were in genes associated with migration and angiogenesis, suggesting that cancer cells suspended in hydrogel may experience a more hypoxic, nutrient deficient environment. Cancer cells heterogeneity also increased under these conditions where more cells lost epithelial landmarks and may have differentiated into various EMT state (15%). Recent studies have suggested that cancer cell within tumors acquire and maintain a diverse array of subpopulations that correspond to transitional hybrid E/M cell states, in vivo [24,25,27,28,29] opposing the classical understanding of EMT as a binary process where cancer cells are either epithelial or mesenchymal [30]. While we did determine that all culturing approaches investigated possessed subpopulations of cancer cells in all previously described EMT states, in vitro cultures promoted a binary-like distribution where the majority of cells were found either in an epithelial or a fully mesenchymal cell state (97% of 2D and 91% of 3D).

Unlike cancer cells cultured in vitro, characterization of cancer cells from in vivo tumors has revealed a high degree of heterogeneity. Epithelial tumors have been shown to exhibit significant plasticity that allowed cancer cells to dynamically shift from epithelial to mesenchymal phenotypes and reside in a range of transitional states. In 2018, Pastushenko et al. highlighted the presence of EMT transition states in a genetic mouse model of squamous cell carcinoma, an epithelial malignancy, and through molecular characterization developed a cell sorting panel for early and late hybrid states to distinguish specific subpopulations actively undergoing EMT. Using the same FACS cell sorting approach developed by Pastushenko et al., our experiments show that in vivo tumors initiated by 4T1 cells produced an EMT profile similar to that found in 10% of squamous cell carcinoma tumors. [24]. The main difference between in vivo cancer cell heterogeneity and that of in vitro or ex vivo cultures was the significant shift towards mesenchymal fate, in vitro, where only ~6% of a cancer cells in BALB/c tumor resided in a mesenchymal state, while 28%–48% cancer cells were mesenchymal, in culture. It is important to note that ex vivo cultured tumoroids also rapidly transitioned to mesenchymal, expanding from 6% in the original tumor, to as much as 48% in the EV3D tumoroid. One potential contributor to this rapid shift to mesenchymal state may be the depletion of a stromal cell type that normally inhibits epithelial to mesenchymal transition. Since neutrophils and inflammatory macrophage populations all seems to rapidly diminish in ex vivo cultured tumoroids, these cell types are likely to contribute to this phenotype.

The deviation of oxygen levels from physiological (~5% in normal tissue and <2% in tumors) to in vitro cultures (21%) may also play a critical role in tumoroid, in vitro behavior [31]. Reactive oxygen species that result from oxidative stress have been linked to numerous cancer types and activation of inflammatory signaling in tumors [32]. The underrepresentation of inflammatory cell populations discovered in this study may a direct response to a reduced oxidative stress imparted by culturing under normoxic conditions. Furthermore, cell signaling pathways up-regulated in tumors (interferon, MAPK, TNF cytokine/interleukin) have previously been linked to oxidative stress responses [32]. Future in vitro tumor platforms should, therefore, be cognizant of oxidative stress and their downstream effects on cellular behavior and tissue dynamics.

It is important to note that NSG mice, similar to fully immune-competent mice, also had a low percentage of cancer cells residing in the mesenchymal state, and NSG mice have normal functioning neutrophils. Therefore, a future testable hypothesis would be to investigate the role neutrophils may have in modulating EMT states. While we clearly find changes in EMT subpopulation ratios as culture conditions are changed, future experiments will have to validate the role these transition states may play in tumor progression and metastasis. Pastushenko et al. suggested that a hybrid EMT phenotype may be associated with increased tumor stemness, whereas a fully epithelial or fully mesenchymal phenotypes may be associated with loss of stem cell markers and tumorigenicity [23]. While this may be the case for some tumor subtypes, additional experiments will have to conducted where hybrid EMT subpopulations would need to be further functionally characterized to determine which tumor behavior they promote.

Direct comparisons of tumors cultured in NSG and BALB/c mice allowed us to determine the impact a fully competent immune system has on the transcriptional program of cancer cells. A thorough understanding is critical for assessing the limitations of characterizing human tumors in PDX mice. As anticipated, we found that cancer cells purified from BALB/c tumors expressed very high levels of cytokines, growth factors and members of the INFα/β and INFγ signaling pathways, hallmarks of an active interaction with the immune system. While PDX mice will continue to be invaluable resources for exploring therapeutic treatments that are independent of immune function, such as chemotherapies and cell cycle checkpoint inhibitors [33], therapies that engage the immune system to kill the tumor will require the development of ex vivo bioreactor type methodologies that will permit the incorporation of all immune components.

Alterations in cancer cell behavior under different growth conditions underlie the importance of defining culturing conditions that preserve endogenous tumor behavior. While monolayer cultures promote the most non-native behavior of cancer cells, this model still maintains value due to its ease and scalability towards applications targeting cancer-growth driving pathways. However, analysis and discovery of potential therapeutics targeting stromal interactions, ECM development, or cell signaling may yield erroneous results in this system. Increased efforts to mimic the complexity of the native tumor microenvironment will be necessary to create useful models used for ex vivo therapeutic screenings and new drug developments. The 3D culturing and inclusion of stromal cell types do show increased similarity to in vivo cancer behavior. However, improvements upon ex vivo culture conditions that allow all stromal components to persist will greatly enhance our ability to conduct pre-clinical screens that may more closely recapitulate the biological responses of patients.

While cancer cell heterogeneity recapitulation will be a necessary method for assessing pre-clinical cancer model efficacy, it is important to note that we only surveyed in vitro cultures and in vivo tumors at a single time point. An ideal tumor model will be capable of reproducing the dynamics of cancer progression, as it is likely that these populations will shift as the cultures/tumors progress, especially during metastasis. Future longitudinal surveys of tumor subpopulations will be required to functionally define the role of these EMT states during cancer progression and metastasis. Additionally, the location of these EMT subpopulations within the tumor microenvironment may be important factors in recapitulating native cancer cell behavior, and a thorough understanding of the tumor architecture will inform any future bioprinting approaches. Subsequent analysis of spatial organization or co-localization with fibroblasts, endothelial and immune cells and how microarchitecture contributes to EMT state will add further resolution to understanding tumor microenvironment effects on cancer cells and be able to educate future tumor model approaches to appropriate cellular compositions. Importantly, this study demonstrates the need for comparative studies which incorporate multiple culture platform types for characterizing pre-clinical cancer model efficacy.

## 4. Materials and Methods

### 4.1. The 2D and 3D Cell Culture

The 4T1 cells were acquired from the American Type Culture Collection (Manassas, MA, USA), are syngeneic and form tumors in immune-competent BALB/c mice. Monocultures, spheroids and 3D gel culture of 4T1 cells were maintained in RPMI Medium 1640 containing 10% FBS with 100,000 U/L of penicillin and 100 mg/L of streptomycin at 37 °C with 5% CO_2_. For monolayer 4T1 RNA collection, 400 cells were seeded into 96-well flat-bottom plates. Aggregates of 4T1 cells were created by incubating 400 4T1 cells into non-adherent U-bottom 96-well plates (Griener, Germany) in 150 µL of complete RPMI containing 0.25% (wt/vol) of caboxymethylcellulose (Sigma Aldrich, St. Louis, MO, USA) for 4 days. On Day 4, spheroids were cast into hydrogels composed of 7.5 % (wt/vol) Type A gelatin from porcine skin (Sigma) and 1% (wt/vol) fibrinogen (Sigma) with thrombin and transglutaminase as crosslinking agents for a final concentration of 100 spheroids/mL of gel solution. Unencapsulated spheroids were cultured in 96 non-adherent U-bottom well plates. Both spheroids encapsulated in gel and unencapsulated spheroids were cultured for an additional 7 days submerged in complete media (Figure 1A). Replicates were isolated from cultures from at least two independent experiments. For imaging, unencapsulated spheroids and spheroids in gels were fixed using 4% paraformaldehyde for 30 min, followed by 3 washes in PBS for 5 min each. Spheroids were stained using phalloidin and DAPI and imaged using a Zeiss LSM 700 confocal microscope.

### 4.2. The 4T1-BFP Generation

The 4T1 cells were transfected using Lipofectamine 3000 (Invitrogen/Thermo Fischer Scientific, Waltham, MA, USA) with the ptagBFP-C plasmid (Evrogen/Axxora, Farmingdale, NY, USA) following manufacturer’s protocol for 48 h. Stable transfected cells were selected with 0.5 mg/mL of G418 for 7 days, followed by FACS sorting for high BFP expression then an additional sort was performed following 7 days of cell expansion in selection media. The 4T1-BFP cells were then inoculated into BALB/c mice until tumors formed, and then tumors were resected, dissociated and then expanded in selection media followed by a final sort for BFP expression. Sublines were established and used in subsequent in vivo allograft experiments.

### 4.3. Allograft Generation and Tumor Digests

All animal experimental procedures were completed under an approved Institutional Animal Care and Use Committee (IACUC) protocol at Lawrence Livermore National Laboratory (LLNL) and conforming to the National Institute of Health (NIH) guide for the care and use of laboratory animals. Female mice (6–8 weeks old) NOD.*Cg-Prkdc^scid^Il2rg^tm1Wjl^*/SzJ (NSG) or BALB/c mice (Jackson Laboratories, Bar Harbor, ME, USA) were injected with 25,000 cells into either mammary fat pad (MFP) or subcutaneously (SQ) into the back flank to establish tumors (N = 5–10 mice per group) in 100 µL of 50% PBS and 50% Matrigel (Corning, Corning, NY, USA). All experimental replicates were generated from at least two independent cohorts. Mice were euthanized 19–26 days post-injection then tumors were dissected. Terminal tumor volume ranged from 70 to 140 mm^3^. Single-cell suspensions of tumor cells were prepared by passing the tumor through a syringe without a needle followed by a 1 h digest with shaking at 37 °C in 100 µg/mL DNase I (Roche, Basel, Switzerland; catalog no. 11284932001), 300 U/mL collagenase/100 U/mL hyaluronidase (Stemcell Technologies, Vancouver, Canada; catalog no. 07912), 0.6 U/mL Dispase II (Roche, Basel, Switzerland; catalog no. 4942078001) in DMEM/D12 with 10% FBS (Gibco, Waltham, MA, USA). Digests were filtered through a 100 µm cell strainer prior to debris removal (Miltenyi Biotec, Bergisch Gladbach, Germany; catalog no. 130-109-398) and resuspended in BD FACS Pre-Sort Buffer (BD, Franklin Lakes, NJ, USA; catalog no. 563503).

### 4.4. Histological Sectioning/Staining

Tumors were excised and flash frozen immediately in liquid nitrogen. Frozen tumors were embedded in optimal cutting temperature (O.C.T.) compound (Fisher Healthcare, Hampton, NH, USA), and sectioned into 40 µm slices, which were placed onto Superfrost Plus microscope slides (Fisher Scientific, Waltham, MA, USA) and stored at −80 °C until further use. To stain sections, slides were warmed to room temperature then immersed in PBS with 4% formaldehyde for 15 min. Sections were then immersed in PBS with 0.5% Triton-X for one hour. The samples were then stained with phalloidin for 45 min and washed three times with PBS. Sections were mounted using Fluoroshield mounting medium containing DAPI (Abcam; Cambridge, United Kingdom) then sealed with a coverslip.

### 4.5. Flow Cytometry and Fluorescent Activated Cell Sorting (FACS)

Dissociation of monolayer and spheroid cultures was accomplished using Accutase (Stemcell Technologies, Vancouver, Canada) until single-cell suspensions were achieved. Prior to Accutase treatment, spheroids were released from hydrogels by first mincing gels into ~1 mm fragments, followed by incubation in Collagenase 1 (Gibco, Waltham, MA, USA; catalog no. 17100-017; 2 mg/mL) in complete media shaking at 37 °C for 1–1.5 h or until gel is completely dissolved. Cell Sorting was performed using either a FACSMelody (BD Biosciences, San Jose, CA; USA) or FACSAria Fusion (BD Biosciences, San Jose, CA; USA) instrument. The following Biolegend antibodies were used at a 1:100 dilution prior to analysis on a BD FACSMelody cell sorter: FITC anti-mouse/human CD11b (ITGAM) (101206), APC anti-mouse CD49d Antibody (ITGA4) (103621), PE/Cy7 anti-mouse β2-microglobulin (154507), FITC anti-mouse CD326 (Ep-CAM) (118207), PE anti-mouse CD51 (104105), Fluor^®^ 647 anti-mouse/rat CD61 (104313), PerCP/Cy5.5 anti-mouse CD106 (105715). Protein expression for in vivo samples was analyzed for 4T1 BFP+ populations only.

### 4.6. RNA Sequencing and Analysis

RLT buffer with β-mercaptoethanol served as the lysis buffer for monolayer, unencapsulated spheroids, and in vivo sorted samples. Spheroids encapsulated in hydrogel and whole tumor samples were lysed using a bead-based homogenization (Lysing Matrix A, MP Bio) in Qiazol (Qiagen, Hilden, Germany). Total RNA was isolated using RNeasy mini spin columns (Qiagen). Sequencing library preparation was performed using Illumina TruSeq RNA Library Preparation Kit v2 (Illumina, San Diego, CA, USA; catalog no. RS-122-2002) and single end 75 base pair sequencing was performed using an Illumina NextSeq 500. Sequencing data quality was checked using FastQC software (https://www.bioinformatics.babraham.ac.uk/projects/fastqc/). Reads were mapped to the mouse genome (mm10) using STAR (version 2.6) [34] and read counts per gene were determined using “featureCounts” from Rsubread package (version 1.30.5; https://bioconductor.org/packages/release/bioc/html/Rsubread.html). Only genes with ≥10 reads in at least 3 samples were selected for analysis. Subsequently, a between-sample normalization was performed using EDASeq (version 2.16.0) [35]. RUVseq (version 1.16.0) was used to estimate the factors of unwanted variation [36]. Differentially expressed genes were identified using edgeR (version 3.22.3), controlling for factors of unwanted variation [37]. A gene was significantly differentially expressed when its false discovery rate adjusted *p*-value was <0.05 and fold change was >2. Gene ontology (GO) and pathway enrichment analysis was performed using ToppGene [38]. Heatmaps were generated using heatmap.2 function in ‘gplots’ R package. Violin plots were generated using ‘ggplot2’ R package. Cytoscape was used for network visualization with pathways defined by Reactome [39,40].

### 4.7. Western Blot

Cell preparations were prepared as previously described. Duplicate samples were pooled then lysed in RadioImmunoPrecipitation Assay (RIPA) buffer followed by centrifuging at 14,000 rcf for 5 min. The supernatants were collected and analyzed using the Jess automated Western blotting system (ProteinSimple, San Jose, CA, USA). Jess reagents (biotinylated molecular weight marker, streptavidin-HRP fluorescent standards, sample buffer, DTT, stacking matrix, separation matrix, running buffer, wash buffer, matrix removal buffer, fluorescent labeled secondary antibodies, antibody diluent, and capillaries) were purchased from the manufacturer and used according to the manufacturer’s standard protocol. Antibodies were diluted with ProteinSimple antibody diluent at the following dilutions: anti Phospho-Cdk1 (Tyr15) (1:12.5, Cell Signaling, Catalog no. 4539), anti-Phospho-MCM2 (Ser139) (1:50, Cell Signaling, Catalog no. 12958), PE/Cy7 anti-STAT1 Phospho (Ser727) (1:12.5, Biolegend, Catalog no. 686407), and β-Tubulin (1:100, Licor, Catalog no. 926-42211). Target protein concentration is quantitated using Compass for SW 4.0 software (https://www.proteinsimple.com/compass/downloads/). The expression of each target protein is normalized to the expression of β-tubulin.

### 4.8. Ex Vivo Tumoroid Culture

The 4T1-Thy1.1 cell line was graciously provided as a gift from Dr. Julian Lum [41] for ex vivo experiments. In total, 2.5 × 10^4^ 4t1-Thy1.1 cells were injected into the MFP of BALB/c mice then cultured to 70–140 mm^3^, isolated and digested as previously described in Allograft Generation and Tumor Digests. Following debris removal of tumor digests, 10,000 cells were plated into a well of a 96-well flat-bottom cell culture plate or a non-adherent U-bottom 96-well plate (Griener, Germany) in 150 µL of complete RPMI. Following 5 days of culture, cells were dissociated with Accutase then either sorted prior to cancer cell RNA isolation, analyzed via flow cytometry, or processed for single-cell RNA sequencing. Cancer cell identification was performed using anti-CD90.1-VioBlue antibody (Miltenyi Biotec, Bergisch Gladbach, Germany; catalog no. 130-102-637).

### 4.9. Single-Cell Sequencing and Data Analysis

Tumor growth, digestion, and isolation of cell suspensions were prepared as previously described for tumor digests and tumoroids were dissociated as previously described. Then, 2 subsequent washes in sterile PBS + 0.04% non-acetylated BSA were performed to further remove debris from final suspension. Cell pellets were resuspended in PBS with 0.04% non-acetylated BSA prior to single-cell sequencing preparation using Chromium Single-cell 3ʹ GEM, Library & Gel Bead Kit v3 (10× Genomics, Catalog no. 1000075) on a 10× Genomics Chromium Controller following manufacturers protocol.

Sequencing data was demultiplexed, quality controlled, and analyzed using Cell Ranger (10× Genomics) and Seurat [42]. Data analysis, expression values, and representative plots were generated using Loupe Cell Browser (10× Genomics) and Seurat (26). The Cell Ranger Single-Cell Software Suite was used to perform sample demultiplexing, barcode processing, and single-cell 3′gene counting. Samples were first demultiplexed and then aligned to the mouse genome (mm10) using “cellranger mkfastq” with default parameters. Unique molecular identifier (UMI) counts were generated using “cellranger count”. Further analysis was performed in R using the Seurat package. For in vivo and ex vivo samples, we performed an integrated analysis to identify and compare common cell types. Cells with fewer than 500 detected genes per cell and genes that were expressed by fewer than 5 cells were not included in the analysis. Prior to data integration, we performed a log-normalization and identified the 2000 most variable genes in each dataset. Subsequently, integration anchors were identified and both datasets were integrated to generate a new integrated matrix. The integrated matrix was then scaled to a mean of 0 and variance of 1 and the dimensionality of the data was reduced by principal component analysis (PCA) (30 principle components). Subsequently, a non-linear dimensional reduction was performed via uniform manifold approximation and projection (UMAP) using the first 20 principle components. Then, we used a graph-based clustering approach to cluster cells. We constructed a K-nearest-neighbor (KNN) graph based on the euclidean distance in PCA space using the “FindNeighbors” function and applied Louvain algorithm to iteratively group cells together by “FindClusters” function (resolution = 0.5). A total of 14 clusters were identified in the integrated dataset.

Cell type identification based on high gene expression of the following genes relative to all cells: Cancer: Epcam; Proliferating Cancer: Epcam/Mki67; Fibroblast: Thy1/Dcn; Myofibroblasts: Thy1/Dcn/Acta2; Endothelial: Pecam1/Cdh5; Neutrophils: S100a8/Retnlg; Myeloid: Ptprc/CD14; M2-like Macrophages: Ptprc/CD14/Mrc1/Cd163; Inflammatory macrophages: Ptprc/CD14/Il1b; Proliferating myeloid: Ptprc/CD14/Mki67; T-Cell/NK Cells: Ptprc/Thy1/CD3e/Nkg7; B Cells: Ptprc/CD19/CD79a.

### 4.10. Statistical Analyses

Statistical analyses were performed using GraphPad Prism. Data is presented from at least three biological replicates. One-way ANOVA and post-hoc Tukey’s Test were used to assess statistically significant differences of mean expression values. Results were considered statistically significant for *p* values < 0.05.

## 5. Conclusions

For cancer drugs to fulfill their promise in clinical trials, we need better screening platforms that reliably mimic responses in humans. Towards this goal, a better understanding of tumor heterogeneity and phenotypic plasticity is needed, as well as of ex vivo conditions that maintain this plasticity post-resection. Recent in vivo work, in immune-competent mice, demonstrated that the cancer EMT process is not binary but rather spans a range of intermediate states, and postulated that these cancer cellular phenotypes may specifically promote cancer progression, metastasis and drug resistance. Here, we showed for the first time that that cancer EMT spectra is modulated by a fully competent immune system, such that tumors grown in immune deficient mice had most of the cancer cells in early hybrid state, while tumors in BALB/c mice had >50% of cancer cells in hybrid and late hybrid states. Most significant, however, is the result that in vitro and ex vivo conditions rapidly promote a mesenchymal state, suggesting that any drug screens conducted on organoids may only reflect the phenotype of these cells, which, in vivo, account for less than 10% of all cancer EMT subtypes. Our study also shows for the first time that tumors cultured ex vivo rapidly become depleted of inflammatory macrophages and neutrophils, while the fibroblasts rapidly expand to include primarily the myofibroblast subtype. These results highlight the need to continue to improve 3D culturing conditions to preserve stromal components and EMT diversity, such that future drug screen can identify therapies that will stop cancer in its track.

## Figures and Tables

**Table 1:** Ontologies associated with genes highly expressed in 2D vs. cancer cells isolated from orthotopic and syngeneic 4T1 mouse tumors.

**Key Ontology Terms Associated with Genes Highly Expressed in 2D Compared to SBM**		
**GO ID**	**Term**	**No. of Genes**
*GO:1901605*	alpha-amino acid metabolic process	34
*GO:0007049*	cell cycle	197
*GO:0044770*	cell cycle phase transition	67
*GO:0051301*	cell division	88
*GO:0045333*	cellular respiration	26
*GO:0007059*	chromosome segregation	60
*GO:0006259*	DNA metabolic process	110
*GO:0006281*	DNA repair	56
*GO:0006260*	DNA replication	54
*GO:0032543*	mitochondrial translation	30
*GO:0007005*	mitochondrion organization	101
*GO:0034660*	ncRNA metabolic process	128
*GO:0000280*	nuclear division	88
*GO:0048285*	organelle fission	91
*GO:0009126*	purine nucleoside monophosphate metabolic process	32
*GO:0006220*	pyrimidine nucleotide metabolic process	11
*GO:0042254*	ribosome biogenesis	97
*GO:0016072*	rRNA metabolic process	77
*GO:0006360*	transcription by RNA polymerase I	14
*GO:0006412*	translation	74
*GO:0006399*	tRNA metabolic process	45
**Key ontology terms associated with genes highly expressed in SBM compared to 2D**		
**GO ID**	**Term**	**No. of genes**
*GO:0001525*	angiogenesis	141
*GO:0001775*	cell activation	208
*GO:0007155*	cell adhesion	341
*GO:0016477*	cell migration	307
*GO:0032963*	collagen metabolic process	49
*GO:0060429*	epithelium development	243
*GO:0030198*	extracellular matrix organization	120
*GO:0006955*	immune response	349
*GO:0000165*	MAPK cascade	178
*GO:0023056*	positive regulation of signaling	321
*GO:0012501*	programmed cell death	357
*GO:0045595*	regulation of cell differentiation	358
*GO:0042127*	regulation of cell proliferation	348
*GO:0034097*	response to cytokine	210
*GO:0070848*	response to growth factor	154
*GO:0034341*	response to interferon-gamma	60
*GO:0070482*	response to oxygen levels	83
*GO:1901700*	response to oxygen-containing compound	314
*GO:0048771*	tissue remodeling	56
*GO:0001944*	vasculature development	191

## Supplementary Materials

The following are available online at https://www.mdpi.com/2072-6694/12/3/690/s1, Figure S1: Purification of 4T1-BFP^+^ cells from BALB/c and NSG tumors; Figure S2: Network visualization of enriched pathways, Figure S3: Heat maps of key cancer pathways, Figure S4: Dot blot of genes used to identify cell clusters, Figure S5: Cancer cell expansion in ex vivo culture.
