# Supplementary material for: Comparative Molecular Analysis of Cancer Behavior Cultured In Vitro, In Vivo, and Ex Vivo
Source: Cancers (Basel). 2020 Mar 14;12(3):690. doi: 10.3390/cancers12030690 (PMC7140030; doi:10.3390/cancers12030690)
Supplement: Supplementary file 1 [file cancers-12-00690-s001.pdf]

cancers

Comparative Molecular Analysis of Cancer Behavior Cultured in vitro, in vivo, and ex vivo

Nicholas R. Hum, Aimy Sebastian, Sean F. Gilmore, Wei He, Kelly A. Martin, Aubree Hinckley, Karen R. Dubbin, Monica L. Moya, Elizabeth K. Wheeler, Matthew A. Coleman and Gabriela G. Loots

Supplemental figures

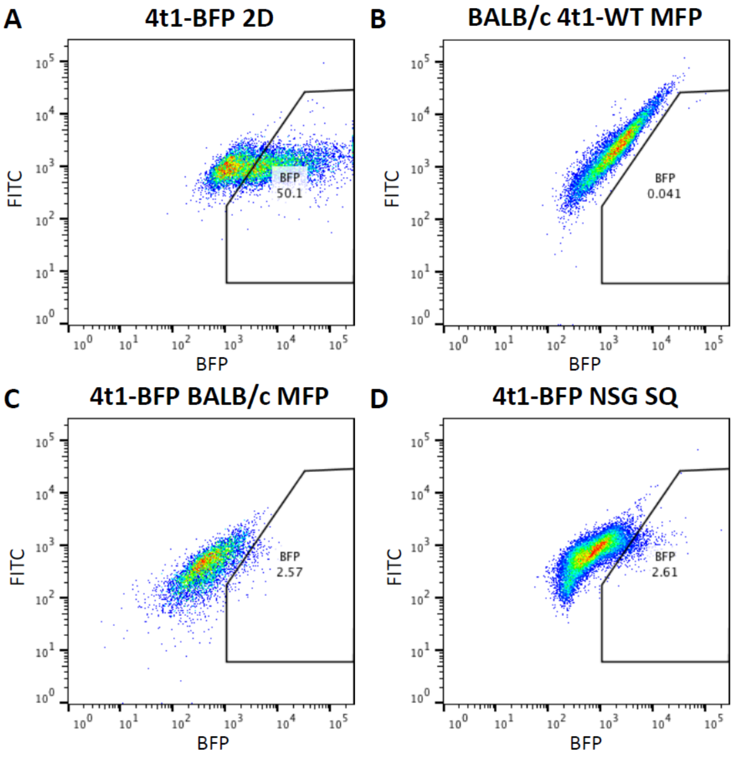

Figure S1. Purification of 4T1-BFP+ cells from BALB/c and NSG tumors. Dot plots of BFP fluorescent intensity of 4T1-BFP+ cells in monolayer culture (**A**). *In vivo* gating of BFP+ tumor cells from a WT 4T1 BALB/c MFP (**B**), 4T1-BFP BALB/c MFP (**C**), or 4T1-BFP NSG SQ (**D**).

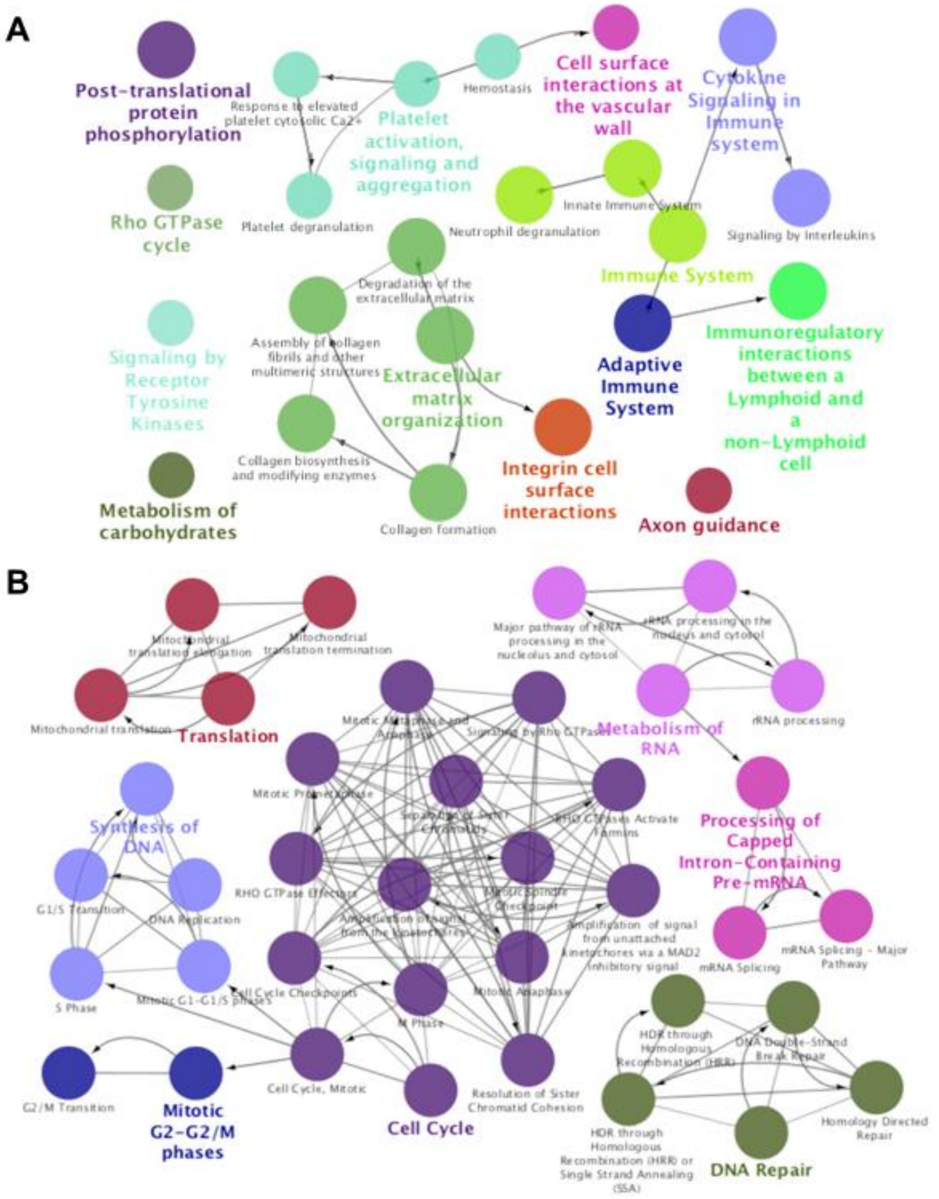

Figure S2. Network visualization of enriched pathways. Pathways enriched in DEGs from 2D culture vs. orthotopic SBM 4T1 culture (**A**). Pathways enriched in DEGs from orthotopic SBM vs 2D culture of 4T1 culture (**B**). Cytoscape was used to generate visualizations with pathways defined from REACTOME.

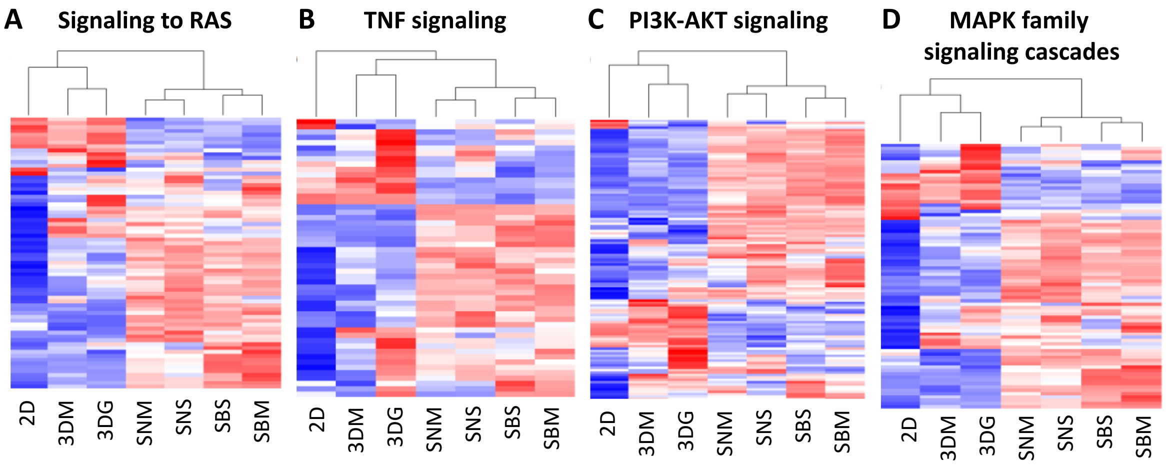

Figure S3. Heat maps of key cancer pathways. Heat map of differentially signaling genes of Ras (**A**), tumor necrosis factor alpha (TNFα) (**B**), phosphoinositide-3 kinase (PI3K) (**C**), and mitogen-activated protein kinases (MAPK) (**D**) signaling pathways. Expression values represented are average of 4-5 replicates.

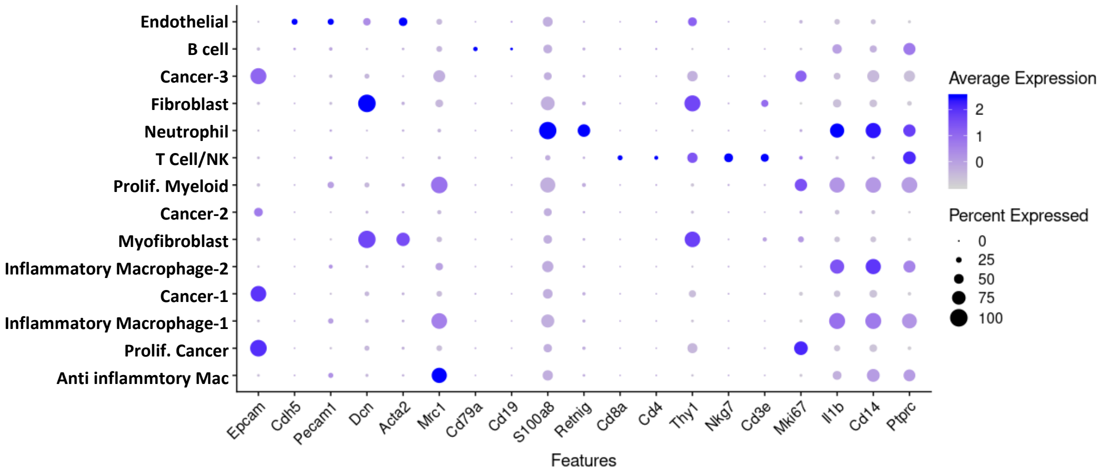

Figure S4. Dot blot of genes used to identify cell clusters. Intensity of dot indicates average cell expression and size of dot represents percent expressed in cluster.

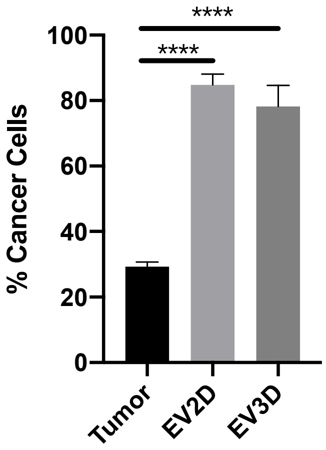

Figure S5. Cancer cell expansion in *ex vivo* culture. Abundance of 4t1-Thy1.1 expressing cells in tumor and 5 day *ex vivo* cultures analyzed via flow cytometry. n = 3 **** $p < 0.0001$.
